# Transcutaneous auricular vagus nerve stimulation augments postprandial inhibition of ghrelin

**DOI:** 10.14814/phy2.15253

**Published:** 2022-04-20

**Authors:** Erica M. Kozorosky, Cristina H. Lee, Jessica G. Lee, Valeria Nunez Martinez, Leandra E. Padayachee, Harald M. Stauss

**Affiliations:** ^1^ Burrell College of Osteopathic Medicine Las Cruces New Mexico USA

**Keywords:** baroreceptor‐heart rate reflex sensitivity, C‐peptide, glucagon, heart rate variability, insulin, RMSSD, SDNN, spectral analysis

## Abstract

Vagus nerve stimulation (VNS) facilitates weight loss in animals and patients treated with VNS for depression or epilepsy. Likewise, chronic transcutaneous auricular VNS (taVNS) reduces weight gain and improves glucose tolerance in Zucker diabetic fatty rats. If these metabolic effects of taVNS observed in rats translate to humans is unknown. Therefore, the hypothesis of this study was that acute application of taVNS affects glucotropic and orexigenic hormones which could potentially facilitate weight loss and improve glucose tolerance if taVNS were applied chronically. In two single‐blinded randomized cross‐over protocols, blood glucose levels, plasma concentrations of insulin, C‐peptide, glucagon, leptin, and ghrelin, together with heart rate variability and baroreceptor‐heart rate reflex sensitivity were determined before and after taVNS (left ear, 10 Hz, 300 µs, 2.0–2.5 mA, 30 min) or sham‐taVNS (electrode attached to ear with the stimulator turned off). In a first protocol, subjects (*n* = 16) were fasted throughout the protocol and in a second protocol, subjects (*n* = 10) received a high‐calorie beverage (220 kCal) after the first blood sample, just before initiation of taVNS or sham‐taVNS. No significant effects of taVNS on heart rate variability and baroreceptor‐heart rate reflex sensitivity and only minor effects on glucotropic hormones were observed. However, in the second protocol taVNS significantly lowered postprandial plasma ghrelin levels (taVNS: −115.5 ± 28.3 pg/ml vs. sham‐taVNS: −51.2 ± 30.6 pg/ml, *p* < 0.05). This finding provides a rationale for follow‐up studies testing the hypothesis that chronic application of taVNS may reduce food intake through inhibition of ghrelin and, therefore, may indirectly improve glucose tolerance through weight loss.

## Graphical abstract

The results of this study show that noninvasive transcutaneous auricular vagus nerve stimulation (taVNS) augments the post‐prandial decline in plasma ghrelin levels. Since ghrelin is the “hunger hormone,” a greater post‐prandial decline in ghrelin is expected to increase satiety, prevent overeating during mealtime, and excessive snacking between meals. These potential effects of taVNS may help overweight or obese patients to lose weight.

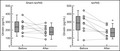

## 1 INTRODUCTION

Recently, highly innovative approaches to control metabolism through neuromodulation, including optogenetic techniques (Fontaine et al., 2021) and focused ultrasound stimulation (Huerta et al., 2021) have been developed. More traditionally, electrical modulation of neurometabolic circuits has been investigated as a potential tool for the treatment of metabolic diseases, including obesity and diabetes (Masi et al., 2018). However, most of these neuromodulatory approaches are invasive (Cigaina, 2004; Vijgen et al., 2013), not available in humans yet (Fontaine et al., 2021; Malbert et al., 2017), or only available in few specialized centers (Huerta et al., 2021). Thus, it is not surprising that cost‐effective and noninvasive alternatives have been explored. Among those, transcutaneous auricular vagus nerve stimulation (taVNS) has been investigated most thoroughly (Farmer et al., 2020), but other noninvasive approaches, such as percutaneous electrical stimulation of dermatome T6 have also been tested for the treatment of obesity (Giner‐Bernal et al., 2020). Regarding taVNS, human cadaver studies demonstrated that the vagus nerve sends afferent sensory nerve fibers to the concha and cymba conchae of the ear (Peuker & Filler, 2002), where the auricular branch of the vagus nerve can be stimulated by taVNS (Butt et al., 2020; Peuker & Filler, 2002). Thus, taVNS selectively stimulates afferent vagal nerve fibers, which is in line with functional MRI studies that demonstrated that taVNS activates the classical vagal centers in the central nervous system, including the nucleus of the solitary tract (Yakunina et al., 2017).

**FIGURE 1:**
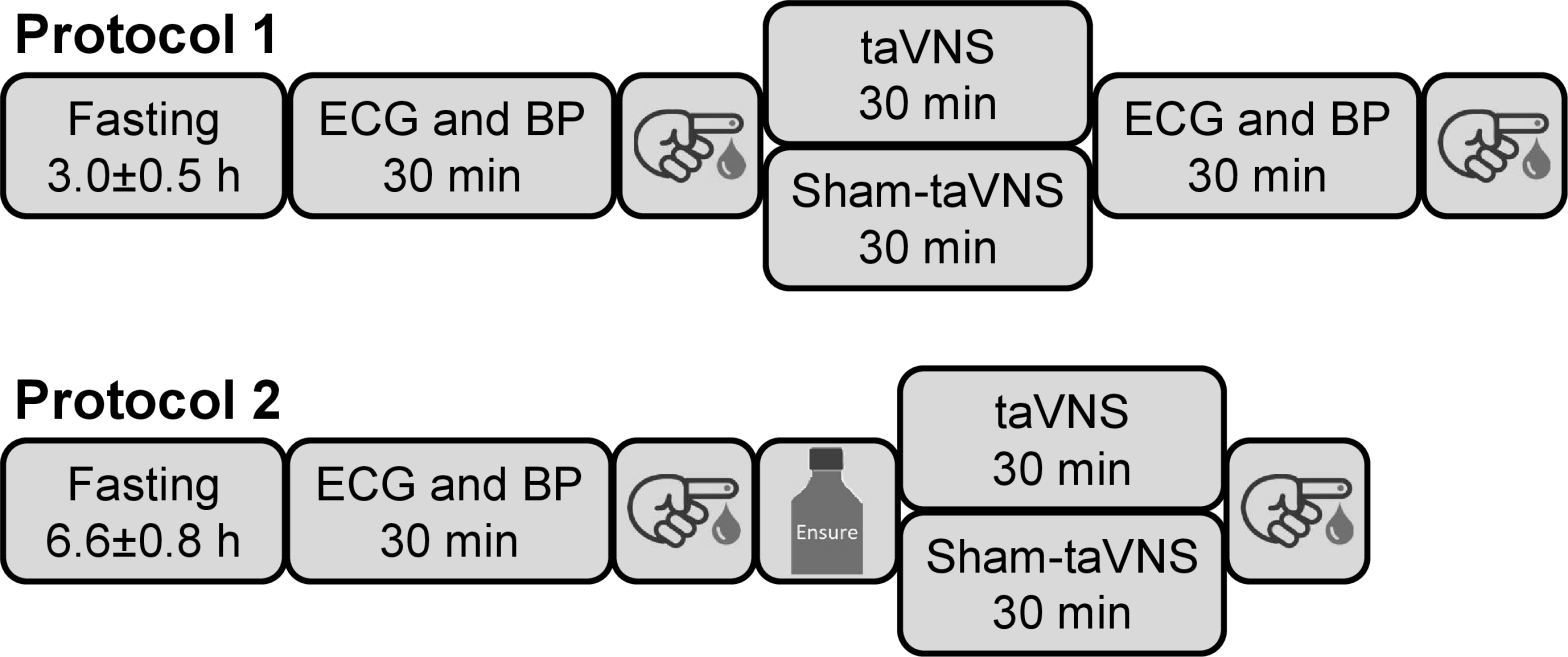
*First protocol*: Subjects reported to the laboratory after an average of 3.0 ± 0.5 h of fasting. The first blood sample was obtained after a 30‐min baseline ECG and blood pressure (BP) recording. Then taVNS or sham‐taVNS (randomized order, at least 1 week apart) was performed for 30 min during which the ECG and BP recordings were continued. This was followed by another 30 min of ECG and BP recording before the second blood sample was obtained. *Second protocol*: Subjects reported to the laboratory after an average of 6.6 ± 0.8 h of fasting. The first blood sample was obtained after a 30‐min baseline ECG and BP recording. Following ingestion of a high‐calorie beverage, taVNS or sham‐taVNS (randomized order, at least 1 week apart) was performed for 30 min during which the ECG and BP recordings were continued. Then the second blood sample was obtained

Our previous studies have demonstrated that cervical VNS inhibits insulin secretion and results in marked elevations in blood glucose levels in non‐diabetic rats even without food intake (Meyers et al., 2016; Stauss et al., 2018). This hyperglycemic response to cervical VNS started almost instantaneously and peak blood glucose levels were observed within less than 30 min (Figure 1 in Meyers et al. (2016)). Depending on the stimulation parameters, cervical VNS may also raise blood glucose levels in non‐diabetic humans (Liu et al., 2020; Stauss et al., 2019). We also demonstrated that this hyperglycemic effect of cervical VNS is mediated through afferent vagal nerve fibers projecting to the central nervous system (Meyers et al., 2016). These findings led to the hypothesis that even in the absence of food intake, acute application of taVNS, through activation of afferent vagal nerve fibers, inhibits pancreatic insulin secretion and raises blood glucose levels, potentially through hepatic glucose release. The first protocol of this study (Figure 1) was designed to test this hypothesis by studying the acute effects of taVNS on blood glucose levels and glucotropic hormones, including glucagon, insulin, and C‐peptide in generally healthy volunteers.

Obesity is closely related to glucose metabolism through its effects on insulin resistance (Wondmkun, 2020). Thus, to obtain a more complete picture of the metabolic effects of taVNS, obesity‐regulating mechanisms, including the hunger/satiety‐regulating hormones ghrelin and leptin (Klok et al., 2007) must also be considered. Chronic cervical VNS has been demonstrated to induce weight loss in patients treated with VNS for depression (Pardo et al., 2007). Importantly, this weight loss was most pronounced in obese patients and achieved without dieting or exercising. In subsequent studies, Bodenlos et al. demonstrated that cervical VNS in patients with major depression acutely alters food cravings and reduces caloric intake and BMI (Bodenlos et al., 2007, 2014). This effect of VNS on food cravings was also reported in an animal study in adult obese minipigs (Val‐Laillet et al., 2010). Since the orexigenic “hunger hormone” ghrelin plays a major role in acute food cravings (Cummings et al., 2001; Klok et al., 2007), we hypothesized that the decrease in plasma ghrelin levels following caloric intake in previously fasted subjects may be augmented by taVNS. The second protocol of this study (Figure 1) was designed to test this hypothesis. In an acute study, we measured ghrelin plasma levels among other metabolic hormones before and after ingestion of a high‐calorie beverage in fasted study participants with concomitant taVNS or sham‐taVNS application. The importance of this protocol is that the application of taVNS before or during a meal may reduce food intake during the meal and, thus, facilitate weight loss in obese patients. Indeed, patients dieting prior to bariatric surgery who concomitantly received percutaneous electrical nerve stimulation of dermatome T6 had lower ghrelin plasma levels and achieved greater weight loss than patients who did not receive the stimulation (Giner‐Bernal et al., 2020). However, to our knowledge, no data on the effects of taVNS on plasma ghrelin levels, especially in the context of food intake are available.

## 2 METHODS

The study was approved by the Institutional Review Board of Burrell College of Osteopathic Medicine (BURRELL IRB 0079_2021) and registered with ClinicalTrials.gov (ClinicalTrials.gov ID: NCT04926415). Two protocols were conducted in generally healthy, non‐diabetic volunteers as illustrated in Figure 1.

### 2.1 First protocol

In a randomized cross‐over design, subjects reported to the laboratory for two study sessions at least 1 week apart. During the experimental study session, taVNS was performed, while a sham‐taVNS procedure was performed during the control study session (randomized order). Subjects were not instructed to be fasted for the study. On average, subjects reported to the laboratory after an average of 3.0 ± 0.5 h of fasting. The study protocol was conducted with the subjects laying in the supine position on examination tables. Pillows and blankets were provided for subjects’ comfort. First, subjects rested for 30 min to establish stable baseline conditions, after which a baseline blood sample was obtained and taVNS or sham‐taVNS was performed for 30 min followed by a recovery period of another 30 min. At the end of the recovery period, a second blood sample was obtained. Throughout the experimental protocol, the ECG was recorded for assessment of autonomic function by heart rate variability analysis, and blood pressure was recorded continuously through noninvasive photoplethysmography (Ohmeda 2300, Finapres) for baroreceptor reflex analysis (Figure 1).

### 2.2 Second protocol

Like in the first protocol, subjects (*n* = 10, 6 female, 4 male) reported to the laboratory for two study sessions at least 1 week apart. During the experimental study session, the study participants received taVNS, while a sham‐taVNS procedure was performed during the control session (randomized order). Subjects were instructed to fast for a minimum of 3 h prior to the study. In average, subjects reported to the laboratory following 6.6 ± 0.8 h of fasting. As in the first protocol, subjects were laying in the supine position on examination tables and pillows and blankets were provided for subjects’ comfort. Following a 30‐min rest period to establish stable baseline conditions, a first blood sample was collected. Then subjects were given a high‐calorie beverage (8 oz of Ensure Original, Abbott Nutritional Products). Subjects could choose between milk chocolate, vanilla, or strawberry flavor. This drink contains 220 kCal, 9 g protein, 33 g total carbohydrates (including 10 g glucose), and 9 g fat. A liquid diet was chosen because it comes in different flavors making it palatable for different tastes, it can be swallowed easily making it convenient for study participants, and because it is individually packed with standardized nutritional values. Following ingestion of the high‐calorie beverage, either the taVNS or the sham‐taVNS procedure was started and maintained for 30 min. Finally, a second blood sample was obtained just before the taVNS or sham‐taVNS application was stopped. Throughout the experimental protocol, the ECG was recorded for assessment of autonomic function by heart rate variability analysis and blood pressure was recorded continuously through noninvasive photoplethysmography (Ohmeda 2300, Finapres) for baroreceptor reflex analysis (Figure 1).

### 2.3 Subjects

The study was performed in generally healthy, non‐diabetic volunteers. The baseline characteristics of the subjects of both protocols are listed in Table 1. Generally, there were more female than male study participants. In average, subjects were normotensive, and heart rate was within normal limits. The average body mass index was in the overweight range. By study design, the fasting duration, defined as the time since the last meal was longer (*p* < 0.05) in the study participants of the second protocol compared to the first protocol. In line with the longer fasting duration, blood glucose levels were lower (*p* < 0.05) in subjects enrolled in the second protocol (Table 1).

**TABLE 1:** Subjects baseline characteristics

Parameter	First Protocol	Second Protocol
*N* (female/male)	16 (13/3)	10 (6/4)
Age (years)	33 ± 4 [24–71, *n* = 16]	44 ± 7 [23–83, *n* = 10]
BMI (kg/m^2^)	27.4 ± 1.6 [17–44, *n* = 16]	25.8 ± 1.9 [17–35, *n* = 10]
Systolic BP (mmHg)	114 ± 3 [95–134, *n* = 16]	116 ± 6 [91–147, *n* = 10]
Diastolic BP (mmHg)	80 ± 2 [72–96, *n* = 16]	80 ± 2 [64–89, *n* = 10]
Heart rate (bpm)	77 ± 3 [46–92, *n* = 16]	74 ± 5 [58–108, *n* = 10]
Fasting duration (hours)	3.0 ± 0.5 [1–9, *n* = 16]	6.6 ± 0.8 [3–10, *n* = 10]*
Glucose (mg/dl)	102.3 ± 1.8 [89–115, *n* = 16]	96.7 ± 2.9 [86–112, *n* = 10]*
Insulin (pg/ml)	828 ± 129 [320–1514, *n* = 9]	146 ± 36 [63–453, *n* = 10]*
C‐Peptide (ng/ml)	3.0 ± 0.3 [1.9–4.5, *n* = 9]	0.6 ± 0.09 [0.3–1.1, *n* = 10]*
Glucagon (ng/ml)	2.2 ± 0.3 [0.9–3.0, *n* = 9]	0.5 ± 0.07 [0.2–0.9, *n* = 10]*
Leptin (ng/ml)	15.3 ± 3.5 [2.3–34.5, *n* = 9]	3.9 ± 1.5 [0.5–15.0, *n* = 10]*
Ghrelin (pg/ml)	668 ± 91 [260–1065, *n* = 9]	259 ± 31 [76–388, *n* = 10]*

Note

Values are pooled baseline data obtained on the first and second study visit (taVNS or sham‐taVNS trial) before the intervention. Data are means ± SEM. Values in brackets are ranges and the number of subjects.

Abbreviations: BM, body mass index; BP, blood pressure; *N*, number of subjects.

* *p* < 0.05 first versus second protocol (independent *t*‐tests).

### 2.4 Transcutaneous auricular vagus nerve stimulation (taVNS)

A bipolar clip electrode with electrode gel applied to the electrode pads was attached to the left ear, so that the cathode was placed at the cymba conchae, next to the external acoustic meatus, and the anode was placed at the back of the ear opposite to the cathode. The bipolar clip electrode was connected to a transcutaneous electrical nerve stimulator (EMS 7500, Current Solutions, LLC). The stimulation parameters were 10 Hz stimulation frequency and 300 µs pulse width. The stimulation current was determined individually for each subject. All subjects were told that they may or may not feel a tingling sensation at the site of the electrode. Then the stimulation current was slowly increased until the subjects indicated that they felt a mild tingling sensation at the site of the electrode or until a maximum current of 2.5 mA was reached. This current was then used for taVNS. All subjects consistently felt the tingling sensation at a stimulation current in a very narrow range of 2.0–2.3 mA. Thus, we are confident that taVNS indeed resulted in activation of sensory nerve fibers at the site of the clip electrode. The stimulation was applied for 30 min.

### 2.5 Sham‐taVNS (control procedure)

Like with the active taVNS, a bipolar clip electrode was attached to the left ear and subjects were told that they may or may not feel the tingling sensation at the ear as the investigators pretended to slowly increase the stimulation current, when in fact the stimulator device was not turned on for the sham‐taVNS intervention. In addition to the stimulator device not being turned on, the electrode cable was also disconnected from the stimulator device. Thus, no stimulation current was applied to the electrode or the ear. Nevertheless, about half of the study participants indicated that they felt a tingling sensation at the ear during the sham‐taVNS application, even though no stimulation occurred. It is possible that these study participants confused the mild pressure sensation associated with the clip electrode being attached to the ear with the tingling sensation that is associated with the real taVNS stimulation. The finding that about half of the study participants reported a tingling sensation with the sham‐taVNS intervention when in fact no current was applied, suggests that the majority of study participants were truly blinded to the intervention. As with taVNS, the sham‐taVNS control procedure was applied for 30 min.

### 2.6 Blood glucose

In each of the two study sessions of both protocols, two blood samples were obtained by finger prick. For each finger prick, the first blood drop was discarded (to avoid contamination by interstitial fluid), and the second blood drop was used for blood glucose determination using indicator strips (ReliOn Prime, distributed through Walmart). Subsequent blood drops (100–500 µl) were collected in EDTA‐covered blood collection tubes. Blood was kept on ice, centrifuged (cooled centrifuge), and plasma stored at −80°C until further processing.

### 2.7 Plasma hormone levels

For determination of plasma hormone concentrations, a Bio‐Plex Multiplex Immunoassay (Bio‐Plex Pro Human Diabetes 10‐Plex Assay, 171A7001 M, Life Science) was used. This assay simultaneously measures plasma concentrations of insulin, glucagon, C‐peptide, glucagon‐like peptide‐1 (GLP‐1), gastric inhibitory peptide (GIP), ghrelin, leptin, plasminogen activator inhibitor‐1 (PAI‐1), resistin, and visfatin from as little as 12.5 µl of plasma (25 µl for measurements in duplicate, as done in this study). For the purpose of this study, we were primarily interested in the glucotropic hormones glucagon, insulin together with its longer half‐life‐byproduct C‐peptide, GLP‐1, and GIP. Because taVNS has been demonstrated to attenuate weight gain in an animal model of obesity (Yu et al., 2021), we were also interested in the hunger/satiety‐regulating hormones leptin and ghrelin (Klok et al., 2007). All blood samples were kept on ice until plasma was separated from blood (cooled centrifuge) and then stored in a −80°C freezer. Nevertheless, the plasma levels of the incretins GLP‐1 and GIP were below the detection threshold of the Bio‐Plex assay, which may be related to their very short half‐lives and the absence of a DPP‐4 inhibitor in the blood collection tubes. Thus, we will only report plasma concentrations for glucagon, insulin, C‐peptide, leptin, and ghrelin.

### 2.8 Heart rate variability analysis

Throughout the study protocols, the ECG was continuously recorded at a sampling rate of 500 Hz. These ECG recordings were divided into 30‐min long sections for the baseline recording (before taVNS or sham‐taVNS), the intervention (taVNS or sham‐taVNS), and the recovery recording following taVNS or sham‐taVNS (only for first protocol). From these 30‐min ECG recordings, beat‐by‐beat heart rate time series were extracted using the Analyzer module of the freely available HemoLab software (Stauss, 2008). From these 30‐min beat‐by‐beat heart rate time series, stationary and artifact‐free segments of 10 min duration were manually selected for further analysis based on visual inspection. The reduction to 10 min was necessary because occasionally movement artifacts and the presence of premature ventricular contractions (PVCs) prevented us from using the complete 30‐min time series. In addition, it usually took 5–10 min at the beginning of each 30‐min section to reach stable (stationary) recording conditions. All further analysis was performed using the Batch Processor module of the HemoLab software (Stauss, 2008). Time and frequency domain heart rate variability analysis was performed as recommended by the Task Force of the European Society of Cardiology and the North American Society of Pacing and Electrophysiology (1996). For time‐domain heart rate variability analysis, the standard deviation of the normal‐to‐normal intervals (SDNN) and the square root of the mean of the sum of the squares of differences between adjacent normal‐to‐normal intervals (RMSSD) were calculated from the 10‐min beat‐by‐beat heart rate time series (50% overlapping segments of 5 min duration). For frequency‐domain heart rate variability analysis, the 10‐min beat‐by‐beat heart rate time series were spline interpolated to a sampling rate of 12 Hz. These equidistantly sampled time series were then used for power spectral analysis using the fast Fourier transform (FFT) algorithm (2048 data values with 50% overlapping segments). Absolute spectral powers were calculated as areas under the curve of the power spectra for the low frequency (LF, 0.05–0.15 Hz), and high frequency (HF, 0.15–0.35 Hz) bands.

### 2.9 Baroreceptor‐heart rate reflex analysis

Baroreceptor‐heart rate reflex analysis was performed using the Analyzer module of the Hemolab software. This software implements the algorithm originally described by Bertinieri et al. (1985) and validated thereafter (Laude et al., 2004; Stauss et al., 2006). Noninvasive blood pressure waveforms obtained by photoplethysmography (Ohmeda 2300, Finapres) were recorded at a sampling rate of 500 Hz. In these blood pressure waveform time series, sequences of a minimum of four consecutive heartbeats where systolic blood pressure and pulse interval continuously increased or decreased were identified. No delay between blood pressure and pulse interval was used because the premise of this study was that taVNS would affect baroreceptor‐heart rate reflex sensitivity through the parasympathetic nervous system that is fast enough to modulate sinus node function within a single heartbeat (Stauss et al., 1997). The slope of the pulse interval over blood pressure relationship was calculated for each individual sequence. The average of these slopes was used as a measure of baroreceptor‐heart rate reflex sensitivity.

### 2.10 Statistical analysis

All data are presented as means ± SEM. For comparison of data obtained in the two study protocols (Table 1), independent *t*‐tests were used. For comparisons of blood glucose levels, plasma hormone concentrations, and hemodynamic data (protocol 2) before and after taVNS or sham‐taVNS, paired *t*‐tests were used. For comparisons of hemodynamic data obtained in the first protocol, a one‐way analysis of variance (ANOVA) for repeated measures (baseline, intervention, recovery) with post hoc Scheffé tests were performed. Statistical significance was assumed for *p* < 0.05.

## 3 RESULTS

### 3.1 Subject characteristics

Study participants were generally healthy with no statistically significant differences in age, body mass index, blood pressure or heart rate between the first and second study protocols. However, most subjects in the first protocol were female (13/16), while gender was more evenly distributed for the second protocol. By study design, participants in the second protocol were fasted for a longer duration compared to subjects in the first protocol. Consequently, baseline blood glucose levels and plasma concentrations of insulin and C‐peptide were significantly lower in participants of the second protocol. Baseline plasma concentrations of glucagon were higher in the first protocol compared to the second protocol, which may be related to the higher proportion of female participants in the first protocol (Horie et al., 2018). Likewise, the higher baseline plasma concentrations of leptin and ghreslin in study participants of the first protocol may be related to gender differences (Hellström et al., 2000; Makovey et al., 2007) (Table 1).

### 3.2 Effects of taVNS on glucose metabolism without caloric intake

**FIGURE 2:**
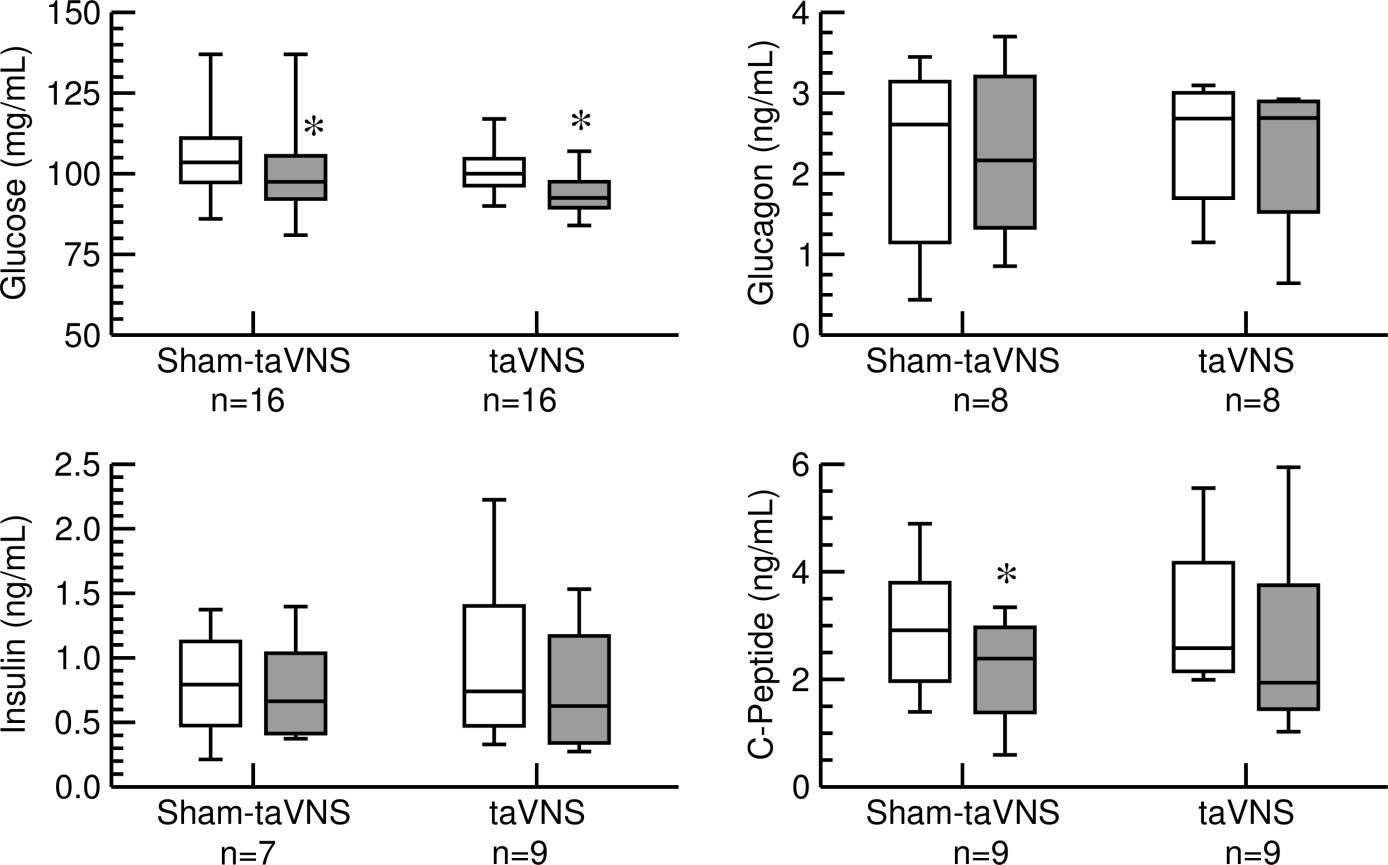
Effects of sham‐taVNS and taVNS on concentrations of blood glucose (top left), plasma glucagon (top right), plasma insulin (bottom left), and plasma C‐peptide (bottom right) were measured in the first protocol without caloric intake. Concentrations before (white columns) and after (grey columns) the taVNS or sham‐taVNS interventions are shown. Data are presented as box‐whisker plots (median, upper/lower quartile, upper/lower extreme). *: *p* < 0.05 before versus after intervention (paired *t*‐test)

Without caloric intake (Figure 1, protocol 1), blood glucose levels declined during the 60 min between the first and second blood draw with no significant difference in the taVNS versus sham‐taVNS trial (Figure 2, upper left). There were no significant differences in glucagon (Figure 2, upper right) and insulin (Figure 2, lower left) plasma concentrations between the first and second blood draw and between the taVNS and sham‐taVNS trials. However, C‐peptide plasma concentrations (Figure 2, lower right) decreased significantly in the sham‐taVNS but not in the taVNS trial. The different results regarding plasma insulin and C‐peptide may be related to the short plasma half‐life of insulin (4–6 min) compared to the long plasma half‐life of C‐peptide (30–60 min). The lack of a decrease in C‐peptide from the first to the second blood draw with taVNS may suggest that taVNS stimulated pancreatic insulin release together with C‐peptide, but the effect of taVNS on insulin may have been masked by its short half‐life (Figure 2).

### 3.3 Effects of taVNS on glucose metabolism in response to caloric intake

**FIGURE 3:**
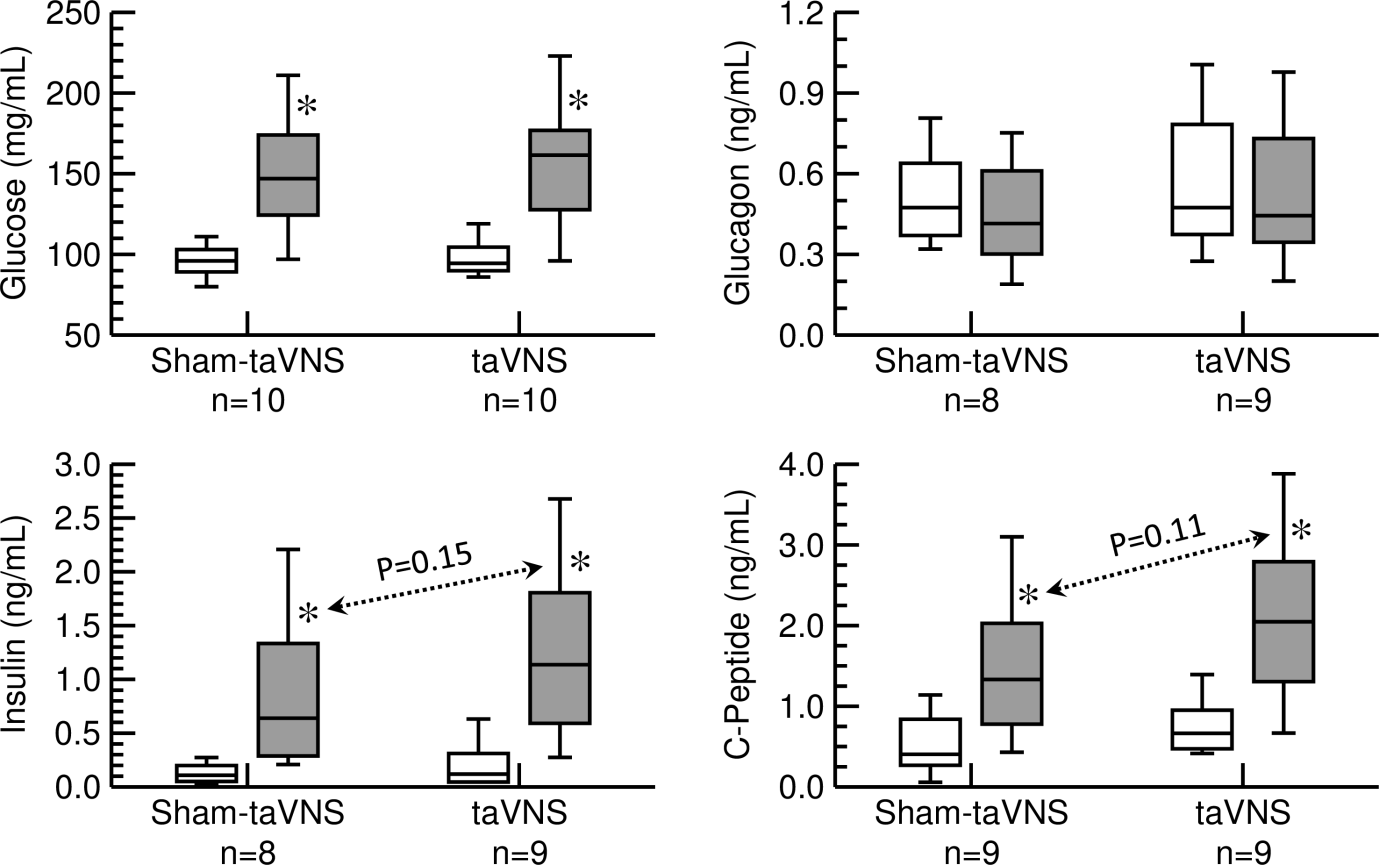
Effects of sham‐taVNS and taVNS on concentrations of blood glucose (top left), plasma glucagon (top right), plasma insulin (bottom left), and plasma C‐peptide (bottom right) in response to ingestion of a high‐calorie beverage (second protocol). Concentrations before (white columns) and after (grey columns) the taVNS or sham‐taVNS interventions are shown. Data are presented as box‐whisker plots (median, upper/lower quartile, upper/lower extreme). *: *p* < 0.05 before versus after intervention (paired *t*‐test)

Thirty min following ingestion of a calorie‐rich beverage (Figure 1, protocol 2) blood glucose concentrations increased significantly independent whether taVNS or sham‐taVNS was applied (Figure 3, upper left). Glucagon plasma concentrations did not change significantly in both the taVNS and sham‐taVNS trials (Figure 3, upper right). As expected after a carbohydrate‐rich beverage, insulin (Figure 3, lower left) and C‐peptide (Figure 3, lower right) increased significantly from the first to the second blood draw. These increases in insulin and C‐peptide were not significantly different for the taVNS (insulin: +1.0 ± 0.2 ng/ml; C‐peptide: +1.3 ± 0.3 ng/ml) and sham‐taVNS (insulin: +0.7 ± 0.2 ng/ml, *p* = 0.17 vs. taVNS; C‐peptide: +0.8 ± 0.2 ng/ml, *p* = 0.12 vs. taVNS) trials, resulting in nonsignificantly elevated postprandial C‐peptide (2.0 ± 0.3 ng/ml vs. 1.4 ± 0.3 ng/ml, *p* = 0.11) and insulin (1.2 ± 0.3 ng/ml vs. 0.8 ± 0.2 ng/ml, *p* = 0.15) plasma levels in the taVNS compared to the sham‐taVNS trial (Figure 3).

### 3.4 Effects of taVNS on hunger/satiety‐regulating hormones

**FIGURE 4:**
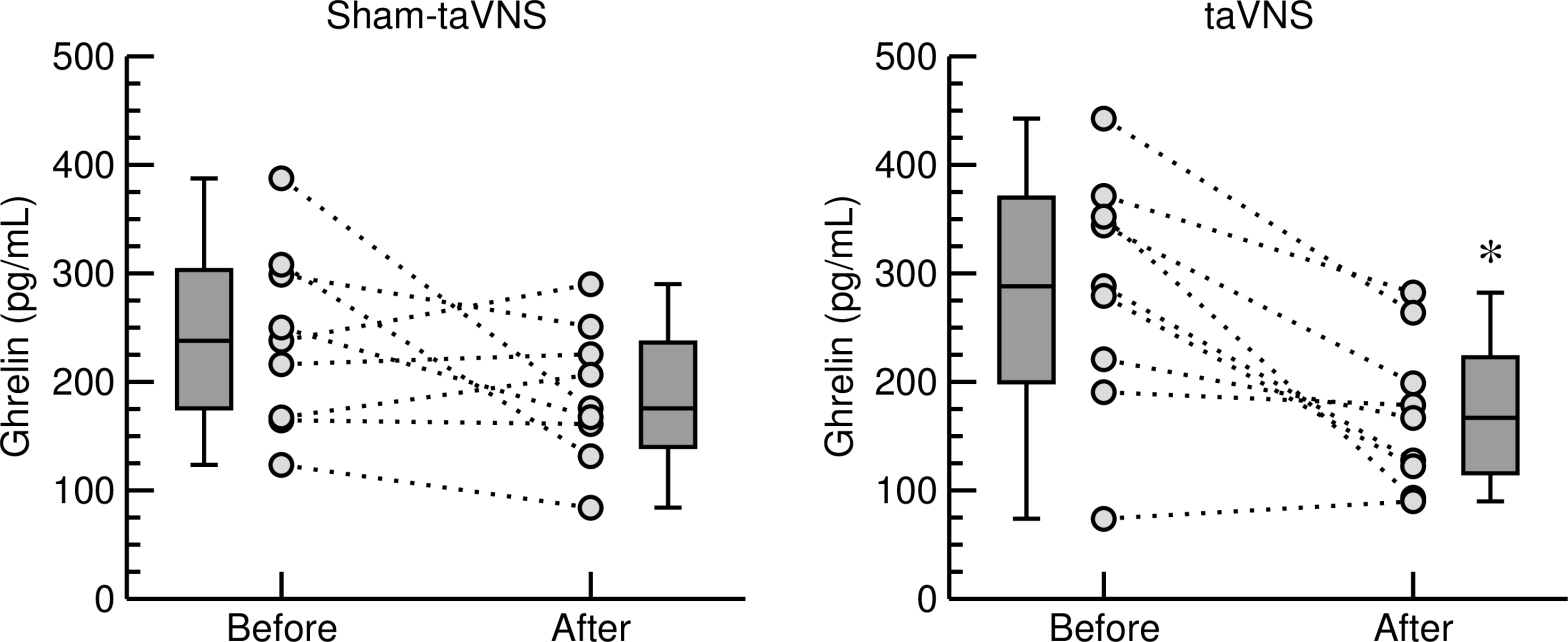
Effects of sham‐taVNS (left) and taVNS (right) on plasma ghrelin concentration in response to ingestion of a high‐calorie beverage (second protocol). Data obtained before and after taVNS or sham‐taVNS are shown for individual study participants (grey circles, *n* = 9) and as box‐whisker plots (median, upper/lower quartile, upper/lower extreme). *: *p* < 0.05 after versus before taVNS (paired *t*‐test)

While leptin is a mediator of long‐term regulation of energy balance, ghrelin is a fast‐acting hormone, playing a major role in meal initiation (Klok et al., 2007). In line with this notion, no significant effects of the acute taVNS or sham‐taVNS interventions on leptin were observed in both protocols (data not shown). Without caloric intake in the first protocol (Figure 1), ghrelin plasma levels did not differ significantly between the first and second blood draw in the taVNS (710 ± 141 pg/ml vs. 725 ± 138 pg/ml) or sham‐taVNS (625 ± 93 pg/ml vs. 772 ± 144 pg/ml) trial. In response to a high‐calorie beverage (second protocol, Figure 1) ghrelin plasma levels did not change significantly in the sham‐taVNS trial with ghrelin levels increasing in three study participants and decreasing in six participants (Figure 4, left). In contrast, in the taVNS trial, plasma ghrelin levels decreased after the high‐calorie beverage in eight from nine study participants (*p* < 0.05) and the one study participant in which ghrelin did not decrease had a very low plasma ghrelin level already before ingestion of the calorie‐rich beverage (Figure 4, right). This significant postprandial decrease in plasma ghrelin levels with taVNS following a carbohydrate‐rich beverage is consistent with anorexic effects of vagal nerve stimulation reported in animal (Chapleau et al., 2016; Stauss et al., 2014; Yu et al., 2021) and human (Bodenlos et al., 2014; Pardo et al., 2007) studies.

### 3.5 Effects of taVNS on heart rate and heart rate variability

**FIGURE 5:**
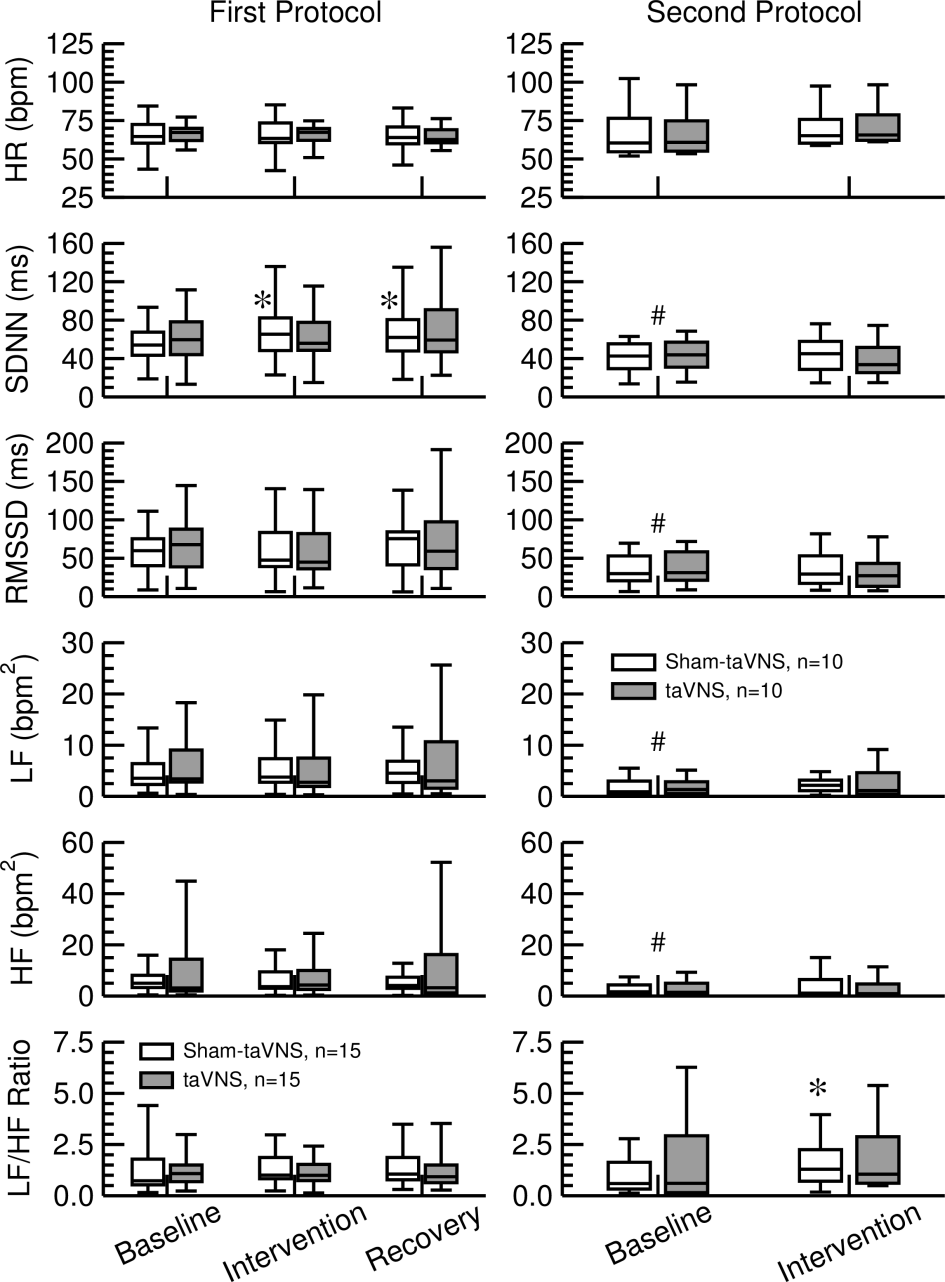
Effect of sham‐taVNS (white bars) and taVNS (grey bars) on heart rate (HR), time‐domain heart rate variability (HRV) parameters standard deviation of NN intervals (SDNN) and root mean square of successive RR interval differences (RMSSD), and frequency‐domain HRV parameters low‐frequency spectral power (LF), high‐frequency spectral power (HF), and LF to HF ratio. The left column shows HR and HRV parameters obtained in the first protocol (*n* = 15) and the right column shows HR and HRV parameters obtained in response to a high‐calorie beverage (second protocol, *n* = 10). Baseline data were obtained during the recording that preceded the taVNS or sham‐taVNS intervention. Intervention data were obtained during the application of taVNS or sham‐taVNS. Recovery data were only recorded in the first protocol and were obtained following the taVNS or sham‐taVNS intervention as illustrated in Figure 1. Data are presented as box‐whisker plots (median, upper/lower quartile, upper/lower extreme). *: *p* < 0.05 versus baseline (1‐way ANOVA with post‐hoc Scheffé test in protocol 1; paired *t*‐test in protocol 2). #: *p* < 0.05 baseline values of second protocol versus baseline values of first protocol (unpaired *t*‐test)

Neither the taVNS nor the sham‐taVNS intervention had any significant effect on heart rate in both study protocols. Heart rate variability (HRV) expressed as SDNN, RMSSD, and LF and HF spectral powers during the baseline recordings before the taVNS or sham‐taVNS interventions was significantly lower in study participants of the second protocol compared to participants in the first protocol. This lower HRV in participants of the second protocol may be related to higher age (+11 years, *p* = 0.16). Interestingly, there were no significant effects of the taVNS intervention (grey bars in Figure 5) on any HRV parameters, including SDNN, RMSSD, LF and HF spectral powers, and LF/HF ratio in both study protocols. Likewise, there were only minor effects of the sham‐taVNS intervention on SDNN (first protocol) and LF/HF ratio (second protocol) with no significant effects on any other HRV parameters. This lack of an effect of taVNS on HRV is consistent with a recent meta‐analysis (Wolf et al., 2021) (Figure 5).

### 3.6 Effects of taVNS on baroreceptor‐heart rate reflex sensitivity

**FIGURE 6:**
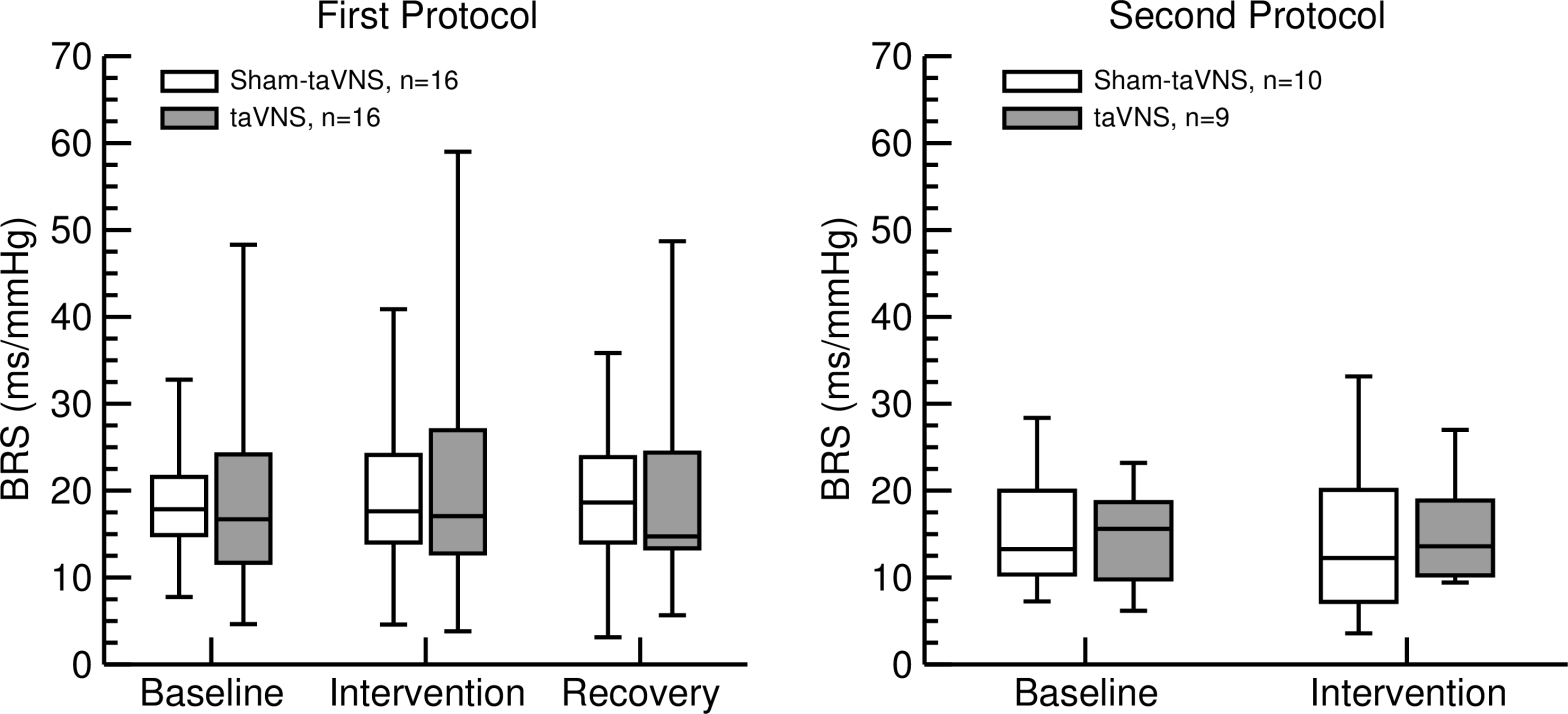
Effects of sham‐taVNS (white bars) and taVNS (grey bars) on baroreceptor‐heart rate reflex sensitivity (BRS) in the first protocol (left) and in response to a high‐calorie beverage (second protocol, right). No significant differences from baseline values were observed during the taVNS or sham‐taVNS interventions (both protocols) or during the recovery recording following the interventions (first protocol only). Data are presented as box‐whisker plots (median, upper/lower quartile, upper/lower extreme)

In both study protocols, neither the sham‐taVNS nor the taVNS intervention had any significant effects on baroreceptor‐heart rate reflex sensitivity. Overall, baseline baroreceptor‐heart rate reflex sensitivity appeared smaller in participants of the second protocol (14.7 ± 1.4 ms/mmHg) compared to participants in the first protocol (18.1 ± 1.6 ms/mmHg, *p* = 0.13), which would be consistent with the slightly higher age (+11 years, *p* = 0.16) in the subjects of the second protocol (Figure 6).

## 4 DISCUSSION

Between meals, plasma levels of the orexigenic peptide ghrelin continuously increase until meal initiation and then fall within 1 h after eating (Cummings et al., 2001). The most important implication of this study is that taVNS appears to augment the normally occurring postprandial decrease in plasma ghrelin levels. This implication is based on the finding that within 30 min following caloric intake, plasma ghrelin levels decreased in 8 from 9 study participants when taVNS was applied (−116 ± 29 pg/ml, *p* < 0.05, Figure 4 right). In contrast plasma ghrelin levels only decreased in 6 of the same 9 study participants when a sham‐taVNS procedure was applied (−51 ± 31 pg/ml, n.sig., Figure 4 left). The significance of this finding is that it provides a rationale for the speculation that taVNS applied before and/or during a meal may reduce caloric intake during that meal and may prevent food cravings between meals due to a more pronounced and potentially longer‐lasting decrease in postprandial ghrelin plasma levels. It is important to note that taVNS did not affect plasma levels of ghrelin in the absence of food intake in protocol 1. Thus, the inhibitory effect of taVNS on ghrelin appears to be linked to food intake and the timing of taVNS relative to food intake appears critical when considering chronic taVNS to facilitate weight loss in obese patients.

The normal physiologic response to caloric ingestion is a decrease in plasma ghrelin levels that reach a minimum at approximately 1 h postprandial (Cummings et al., 2001). This time course is in line with the plasma half‐life of ghrelin of about 30 min (Akamizu et al., 2004). With this regard, it is important to note that the postprandial blood sample was obtained at 30 min postprandial. At this time point, plasma ghrelin levels may not yet have reached their postprandial minimum, which would have been expected at 60 min postprandial (Cummings et al., 2001). The finding that plasma ghrelin levels had already decreased significantly at 30 min postprandial with taVNS but not with the sham‐taVNS intervention further suggests that taVNS indeed augments the physiologic postprandial inhibition of ghrelin.

Another objective of this study was to investigate the potential effects of taVNS on glucose metabolism. In two protocols we studied the acute effects of taVNS on blood glucose levels and glucotropic hormones with and without caloric intake. Without food intake (first protocol) blood glucose levels decreased significantly from the first to the second blood draw, independent of whether taVNS or sham‐taVNS was applied. Because subjects had not eaten for an average of 3 h prior to the study, insulin plasma levels were already low at the beginning of the protocol and did not further decrease by the end of the protocol. Possibly due to the longer half‐life of C‐peptide (30–60 min) compared to insulin (4–6 min), a significant decrease in C‐peptide plasma levels was observed in the sham‐taVNS trial. In contrast, C‐peptide plasma levels did not decrease significantly in the taVNS trial (Figure 2). It is, therefore, possible that C‐peptide together with insulin was released from the islets of Langerhans (1869) during the 30 min of taVNS. During the subsequent 30 min recovery period and before the second blood sample was drawn, insulin levels returned rapidly towards baseline levels, while C‐peptide levels—owing to the longer half‐life—remained at baseline levels. Even though statistically significant, this potential effect of taVNS on pancreatic insulin and C‐peptide release was small and may not have clinical implications. Further evidence for the lack of a clinically relevant acute effect of taVNS on glucose metabolism is provided by the results of our second study protocol (Figure 1). Following caloric intake, the anticipated results, including increases in blood glucose levels and plasma concentrations of insulin and C‐peptide were obtained. While there were trends for higher postprandial plasma insulin (*p* = 0.15) and C‐peptide (*p* = 0.11) levels with the taVNS interventions compared to the sham‐taVNS intervention (Figure 3), these potential effects of taVNS were not statistically significant, further questioning a clinically significant effect of acute taVNS. The finding of a lack of a significant effect of taVNS on glucose metabolism after caloric intake in our study is consistent with a recent study by Vosseler et al. (2020), who studied the effects of taVNS on glucose metabolism in a 2‐h oral glucose tolerance test in exclusively male subjects. Like in our second protocol, no significant effects of taVNS on glucose metabolism were observed by these investigators. Since our study included more female than male subjects, the findings by Vosseler et al. (2020) appear to be not limited to male subjects.

However, the lack of significant acute effects of taVNS on glucose metabolism in generally healthy participants in our study and in the study by Vosseler et al. (2020) does not allow for the conclusion that taVNS is ineffective in patients with type 2 diabetes. For example, Huang et al. (2014) reported improved glucose tolerance following 12 weeks of self‐administration of taVNS in 72 participants with impaired glucose tolerance. The same group of investigators also reported that daily taVNS applications for 34 consecutive days prevented development of hyperglycemia in Zucker diabetic fatty rats and increased insulin receptor expression in various tissues, including liver and skeletal muscle (Li et al., 2014). However, these investigators also reported a substantial decrease in body weight in Zucker diabetic fatty rats with daily taVNS application (Yu et al., 2021). Thus, it is unclear if the chronic effect of taVNS on glucose metabolism in patients with impaired glucose tolerance (Huang et al., 2014) and diabetic animals (Li et al., 2014) observed by these investigators is a direct effect of taVNS on glucose metabolism or an indirect effect caused by improved insulin sensitivity and upregulated insulin receptors secondary to taVNS‐induced weight loss. With this regard, it is possible that taVNS primarily affects food intake, potentially through its effect on postprandial ghrelin levels. Chronically, this effect may result in weight loss and, thus, improved glucose tolerance.

A recent meta‐analysis concluded that “there is no support for the hypothesis that vagally mediated heart rate variability is a robust biomarker for acute taVNS” (Wolf et al., 2021). The results of our time‐ and frequency‐domain heart rate variability analysis confirms this conclusion. In both of our protocols (Figure 1), acute taVNS did not affect heart rate, SDNN, RMSSD, low‐ and high‐frequency spectral power of heart rate, or cardiac autonomic balance expressed as LF/HF ratio (Figure 5). In addition, no effects of taVNS on baroreceptor‐heart rate reflex sensitivity was noticed (Figure 6). However, these negative findings on the effects of taVNS on heart rate variability and baroreceptor‐heart rate reflex function do not mean that taVNS has no effects on autonomic nervous system function. It is possible that taVNS affects autonomic target organs other than the heart, such as the gastrointestinal tract, the liver, or the pancreas. Furthermore, in our current study, we investigated the acute effects of taVNS on cardiac autonomic function. It is possible that the autonomic effects of taVNS only become apparent with repeated chronic applications. For example, in previous studies we applied taVNS on three consecutive days (Dalgleish et al., 2021; Kania et al., 2021). As in the current study, no significant effects of taVNS on heart rate variability were observed on the first study day of these prior studies. However, when pooling the data from all three study days, a significant increase in RMSSD was observed following taVNS, suggesting that repeated chronic applications of taVNS increases parasympathetic modulation of cardiac function.

The mechanisms by which taVNS inhibits postprandial ghrelin were not directly addressed in our study. However, it is noteworthy that the gastrointestinal synthesis of ghrelin is not only regulated by food intake. For example, ghrelin secretion is stimulated by muscarinic agonists (Hosoda & Kangawa, 2008) and inhibited by GLP‐1 via increased insulin secretion (Hagemann et al., 2007). Thus, it is important to point out that taVNS did not affect heart rate variability (Figure 5) or baroreceptor reflex function (Figure 6), suggesting that taVNS did not alter peripheral efferent parasympathetic tone that otherwise may have stimulated ghrelin secretion via muscarinic receptors (Hosoda & Kangawa, 2008). While not conclusive, the lack of a decrease in C‐peptide with taVNS in the first protocol (Figure 2) and the trend towards higher C‐peptide (*p* = 0.11) and insulin (*p* = 0.15) plasma levels with taVNS in the second protocol (Figure 3) give rise to the intriguing speculation that taVNS may facilitate pancreatic insulin release that then inhibits gastrointestinal ghrelin secretion (Hagemann et al., 2007). However, future studies specifically designed to test this possibility would be necessary before such a conclusion can be made.

Interestingly, baseline values for blood glucose levels, plasma concentrations for insulin, C‐peptide, glucagon, leptin, and ghrelin (Table 1), heart rate variability parameters (Figure 5), and baroreceptor‐heart rate reflex sensitivity (Figure 6) differed between the two protocols even before any interventions were done. Despite similar recruitment efforts, more female subjects were enrolled in the first protocol than in the second protocol (81% vs. 60%). In addition, subjects in the first protocol generally tended to be younger than subjects in the second protocol (33 ± 4 vs. 44 ± 7 years of age, *p* = 0.16). Finally, by study design, the fasting duration was significantly shorter in the first protocol compared to the second protocol (Table 1). The longer fasting period in the second protocol most likely explains the lower blood glucose levels and lower plasma concentrations of insulin and C‐peptide compared to the first protocol. It is known that females—in average—have higher plasma leptin (Hellström et al., 2000) and ghrelin (Makovey et al., 2007) levels compared to males, which is generally assumed to be related to gender differences in body composition. Thus, the higher number of female study participants in the first protocol may explain the higher plasma leptin and ghrelin levels compared to the second protocol. It is known that aging is associated with a decrease in heart rate variability (Garavaglia et al., 2021; Jensen‐Urstad et al., 1997) and baroreceptor‐heart rate reflex sensitivity (Gribbin et al., 1971; Kornet et al., 2005). Thus, the lower heart rate variability (SDNN, RMSSD, LF, and HF spectral power of heart rate, Figure 5), as well as the lower baroreceptor‐heart rate reflex sensitivity (Figure 6) in the participants of the second protocol may be related to higher age.

In conclusion, the results of this study imply that taVNS augments the normally occurring postprandial decline in plasma ghrelin levels. Since the physiological role of ghrelin is to initiate food intake, a stronger postprandial decline in ghrelin plasma levels would be expected to reduce food intake during or in‐between meals. It would be interesting to design future chronic studies to identify the optimal timing of taVNS relative to the time of a meal that results in the lowest food intake during that meal. The availability of taVNS devices the size of a hearing aid (e.g., https://www.coolstim.com/ or https://www.sparkbiomedical.com/) make such studies practical. Another strategy would be to utilize chronic taVNS to suppress food cravings and prevent snacking throughout the day. Preventing snacking in the late evening may be particularly effective for weight loss, because chrono‐nutritional studies have demonstrated that consuming high caloric meals later in the day is associated with obesity (Mazri et al., 2021; Okada et al., 2019). Even though our findings are encouraging, more research including chronic application of taVNS is needed to address issues such as the optimal stimulation parameters or the timing of stimulation relative to food intake, before chronic taVNS can be developed into a clinical tool to assist obese patients with achieving their weight loss goals.

## CONFLICT OF INTEREST

The authors have no conflicts of interest to declare.

## ETHICS STATEMENT

The study was approved by the Institutional Review Board of Burrell College of Osteopathic Medicine (BURRELL IRB 0079_2021) and registered with ClinicalTrials.gov (ClinicalTrials.gov ID: NCT04926415).

## AUTHOR CONTRIBUTIONS

Harald M. Stauss conceived and designed the experiments. All authors performed experiments, analyzed data, and contributed to writing and revising the manuscript. Specifically, Erica M. Kozorosky, Cristina H. Lee, Jessica G. Lee, Valerie Nunez, and Leandra E. Padayachee contributed equally to this study.
